# Undersized telomeres in regulatory T cells link to the pathogenesis of allergic rhinitis

**DOI:** 10.1016/j.isci.2023.108615

**Published:** 2023-12-10

**Authors:** Jinmei Xue, Zhizhen Liu, Yun Liao, Xiwen Zhang, Yu Liu, Lihua Mo, Rui Dong, Qiang Li, Xizhuo Sun, Jun Xie, Pingchang Yang

**Affiliations:** 1 Department of Otolaryngology, Head & Neck Surgery, Second Hospital, Shanxi Medical University, Taiyuan, China; 2 Department of Biochemistry and Molecular Biology, Shanxi Key Laboratory of Birth Defect and Cell Regeneration, Key Laboratory of Coal Environmental Pathogenicity and Prevention, Ministry of Education of China, Shanxi Medical University, Taiyuan, China; 3 Shenzhen Clinical School of Medicine, Guangzhou University of Chinese Medicine, Shenzhen, China; 4 Institute of Allergy & Immunology of Shenzhen University, State Key Laboratory of Respiratory Diseases Allergy Division at Shenzhen University, Shenzhen, China; 5 Department of General Practice Medicine, Third Affiliated Hospital, Shenzhen University, Shenzhen, China

**Keywords:** cell biology, immunology

## Abstract

Telomeres are an important biomarker in the cell destiny. The relationship between telomeres and regulatory T cells (Tregs) has not yet been investigated. The objective of this study is to evaluate the link between Tregs' telomere length and allergic rhinitis (AR)’s pathogenesis. Here, we report that low telomerase activity and high endoplasmic reticulum stress status were observed in Tregs from AR patients, as shown in the results. Immune regulatory molecules levels were correlated with the length of Tregs' telomeres. The immune-suppressive functions of Tregs were associated with the telomere length/Telomerase reverse transcriptase/Telomerase protein component 1 status in Tregs. The levels of telomere length/telomerase in airway Tregs were reduced by sensitization. Endoplasmic reticulum stress signaling pathway of proline-rich receptor-like protein kinase-eukaryotic translation initiation factor 2A (eIF2a) was associated with the regulation of telomerase. Inhibiting eIF2a had an effect on upregulating telomerase activity in Tregs and mitigating experimental AR.

## Highlights

• Immune regulatory molecules are associated with the short length of Tregs' telomeres
• Treg functions are associated with the telomere length and telomerases
• The telomere length and telomerase in Tregs can be reduced by sensitization
• Inhibiting eIF2a increases telomerase activity in Tregs and mitigating AR in mice

## Graphical abstract

**Figure:**
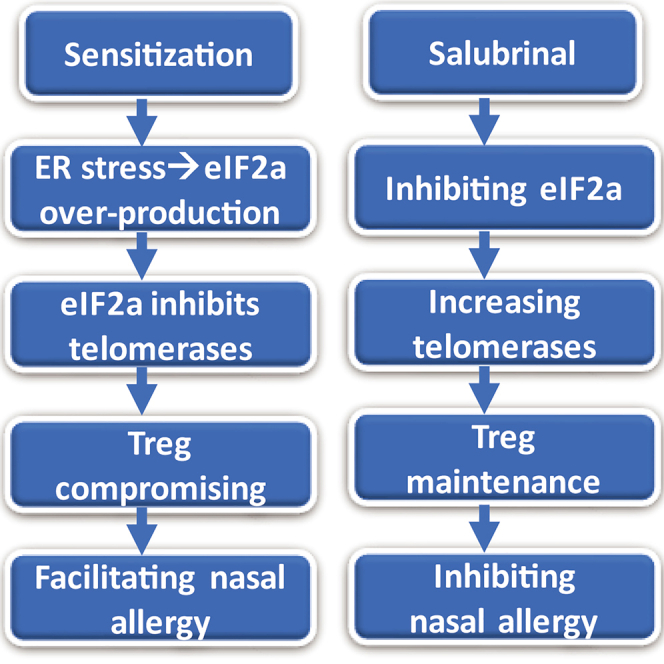

## Introduction

Allergic diseases are adverse reactions of the immune system in the body against the invaded innocent antigens.^1^ Dendritic cells (DCs) are responsible for capturing and processing the invaded antigens. Antigen information and costimulatory molecules stimulate the transformation of naive CD4^+^ T cells into Th2 cells. The primed Th2 cells pass antigen information along with relevant cytokines to B cells, which drive the B cells to become plasma cells. Immunoglobulin E (IgE) is produced and released by plasma cells into the microenvironment. The high-affinity receptors are bindable by IgE to sensitize mast cells. The re-exposure to specific antigens activates the sensitized mast cells.^2^^,^^3^ Thereby, allergy attacks are triggered. The underlying mechanism is still not fully understood.

In general, immune response is tightly regulated by the immune regulatory system in the body.^4^ Regulatory T cells (Tregs) and regulatory B cells (Bregs) are the canonical components of the immune regulatory system. CD4^+^ CD25^+^ Foxp3^+^ Tregs have attracted more attention due to their powerful and effective immune regulatory capacity.^5^ Patients with allergic diseases exhibit a Th2 bias due to the dysfunction of their immune regulatory system.^6^^,^^7^ The mechanism of Treg dysfunction is still awaiting further investigation.

Endoplasmic reticulum (ER) stress was previously reported to have an impact on Treg’s functions.^8^ ER stress indicates a condition where the demand for protein production in the cell increases upon extrinsic stimuli. As a result, increased amounts of unfolded or misfolded proteins accumulate in the ER. This leads to the unfolded protein response in the cell. Many gene transcription activities are initiated in cells to produce specific proteins. The purpose of this is to restore the homeostasis in cells.^9^ However, if the stimuli are too strong or last for too long time, the unwanted protein molecules may be induced. ER stress-related overproduction of X-box binding protein-1 (XBP1) can cause disruption in dendritic cell homeostasis and reduce their ability to fight tumors.^10^ Proline-rich receptor-like protein kinase (PERK) and eukaryotic initiation factor 2-α (eIF2a) are also ER stress-associated molecules, which are involved in regulating many biochemical processes, such as cardiac hypertrophy,^11^ osteoporosis,^12^ arthritis,^13^ and inhibition of tumor necrosis factor alpha (TNF-α)-triggered nuclear factor κB (NF-κB) activation.^14^ However, the detailed mechanism by which eIF2a regulates Treg’s destiny remains unknown.

Published data indicate that aging-related telomere length (TL) is a biomarker of the cell destiny. TL is associated with the pathogenesis of age-related diseases as well as some other diseases.^15^ Telomeres are nucleoprotein structures located at the end of chromosome arms. The primary function of telomeres is to maintain genome stability. Telomeres form a highly conserved, hexameric (TTAGGG) tandem repeat DNA sequence.^16^ TTAGGG is the sequence in humans and mammals while other sequences exist in other organisms. TL can be influenced by number of cell divisions. When the length of TL reduction reaches a critically short length, it can induce a DNA damage response (DDR), which can result in either senescence or apoptosis.^16^^,^^17^ TL is also influenced by events other than aging, such as cancer,^18^ arthritis,^19^ and diabetic nephropathy.^20^ The reduction of Treg numbers has been recognized in subjects with allergic diseases.^21^ Reduction of TL can affect the lifespan of the cell.^15^ It is possible that the shortening of TL may contribute to the reduction of Treg number in subjects with allergic diseases. To test the hypothesis, we collected blood samples from 50 patients with allergic rhinitis (AR) and 50 healthy control (HC) subjects. TL in Tregs was shorter in the AR group, which was positively correlated with the AR-associated parameters. Sensitization increased the activities of eIF2a in Tregs, which disturbed the telomere system and compromised Treg’s functions. Inhibition of eIF2a restored the homeostasis of the telomere system, improved the functions of Tregs, and mitigated experimental AR.

## Results

### Tregs from AR patients show low telomerase activity and high ER stress status

**Figure 1:**
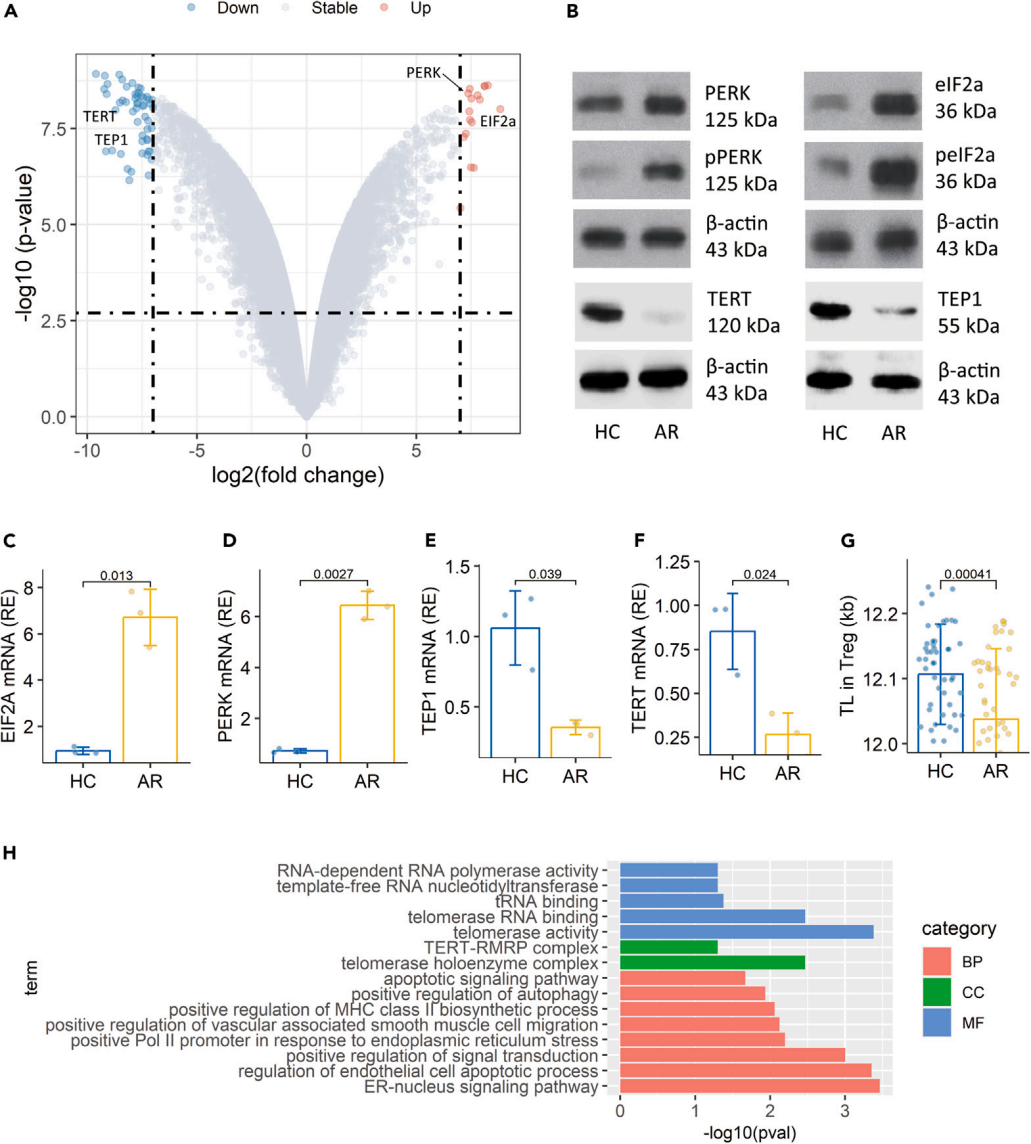
Tregs of AR patients show low telomerase activity, high ER stress status, and shorter TL PBMCs were prepared from blood samples collected from patients with AR (n = 50) and HC subjects (n = 50). Tregs were purified from PBMCs, and analyzed by RNA-seq (A), western blotting (B), and RT-qPCR (C). (A) A volcano plot shows 4 significant DEGs. (B) Immunoblots show protein profile of the 4 DEGs. (C‒F) Boxplots show median (IQR) of the mRNA levels of the 4 DEGs. The data of B and C represent 3 independent experiments with pooled samples of 50 subjects per group. (G) Median (IQR) of TL in Tregs of 50 subjects per group. (H) GO annotation results. Statistics: Mann-Whitney test. Abbreviations: HC: Healthy control. AR: Allergic rhinitis. PBMC: Peripheral blood mononuclear cell. Treg: Regulatory T cell. RNA-seq: RNA sequencing. DEG: Differentially expressed gene. TL: Telomere length. pPERK and peIF2a: Phosphor PERK and phosphor eIF2a. BP: Biological process. CC: Cellular components. MF: Molecular function. GO: Gene oncology.

Tregs were isolated from blood samples collected from 50 AR patients and 50 HC subjects (Table 1). RNA samples were prepared with Tregs and analyzed by RNA sequencing (RNA-seq). In the 60 differentially expressed genes (DEGs), we found 15 upregulated DEGs and 45 downregulated DEGs. Among the DEGs, *PERK* and *EIF2A* were upregulated, while *TERT* (telomerase reverse transcriptase) and *TEP1* (telomerase protein 1) were downregulated (Figure 1A). The changes of DEGs were verified by conventional quantitative reverse-transcription PCR (RT-qPCR) and western blotting (Figures 1B–1F). DNA samples were prepared with Tregs and analyzed by qPCR to measure TL. The results showed that TL was shorter in the AR group than it was in the HC group (Figure 1G). The gene ontology (GO) annotation analysis showed marked difference between the HC group and the AR group in Tregs’ TERT-RMRP complex and telomerase holoenzyme complex, the telomerase activity, and telomerase RNA binding activity. Strong ER-nucleus signaling pathway activity was detected (Figure 1H). The results demonstrate that Tregs from AR patients are at a high ER stress status and implicate that the telomere in Tregs may be disturbed.

**Table 1:** Demographic data of AR patients

Items	AR patients	HC subjects
Number	50	50
Age (years)	24.39 ± 2.72	25.00 ± 2.93
Male (%)	25 (50)	25 (50)
Female (%)	25 (50)	25 (50)
FEV1 (% predicted)	99.41 (96.4, 102.1)	100.2 (96.8,102.2)
Serum IgE (IU/mL)	355.7 ± 33.9	25.2 ± 5.6
Serum sIgE (positive, %)	50 (100)	0
Co-suffer allergy		
Allergic asthma	2	0
Allergic dermatitis	3	0
Food allergy	2	0
Using corticosteroids^a^	50	0
Blood neutrophil (10^9^/L)	5.22 (4.68, 6.19)	5.11 (4.17, 6.09)
Blood eosinophil (10^9^/L)	0.41 (0.19, 0.66)	0.13 (0.10, 0.22)
SPT results		
Mite mix	50 (100%)	0
Timothy grass	3 (6%)	0
Bermuda grass	2 (4%)	0
Pine	5 (10%)	0
Mold mix	3 (6%)	0
Poplar	4 (8%)	0
Rye	2 (4%)	0
Mugwort	3 (6%)	0
Animal dander	4 (8%)	0

The data are presented as means ± SD or median (IQR).

FEV1: Forced expiratory volume in 1 s.

Specific IgE (sIgE) > 0.35 IU/mL was considered as positive.

a Patients used corticosteroid spray to control AR attacks.

### TL in Tregs is associated with the immune regulatory molecule levels

**Figure 2:**
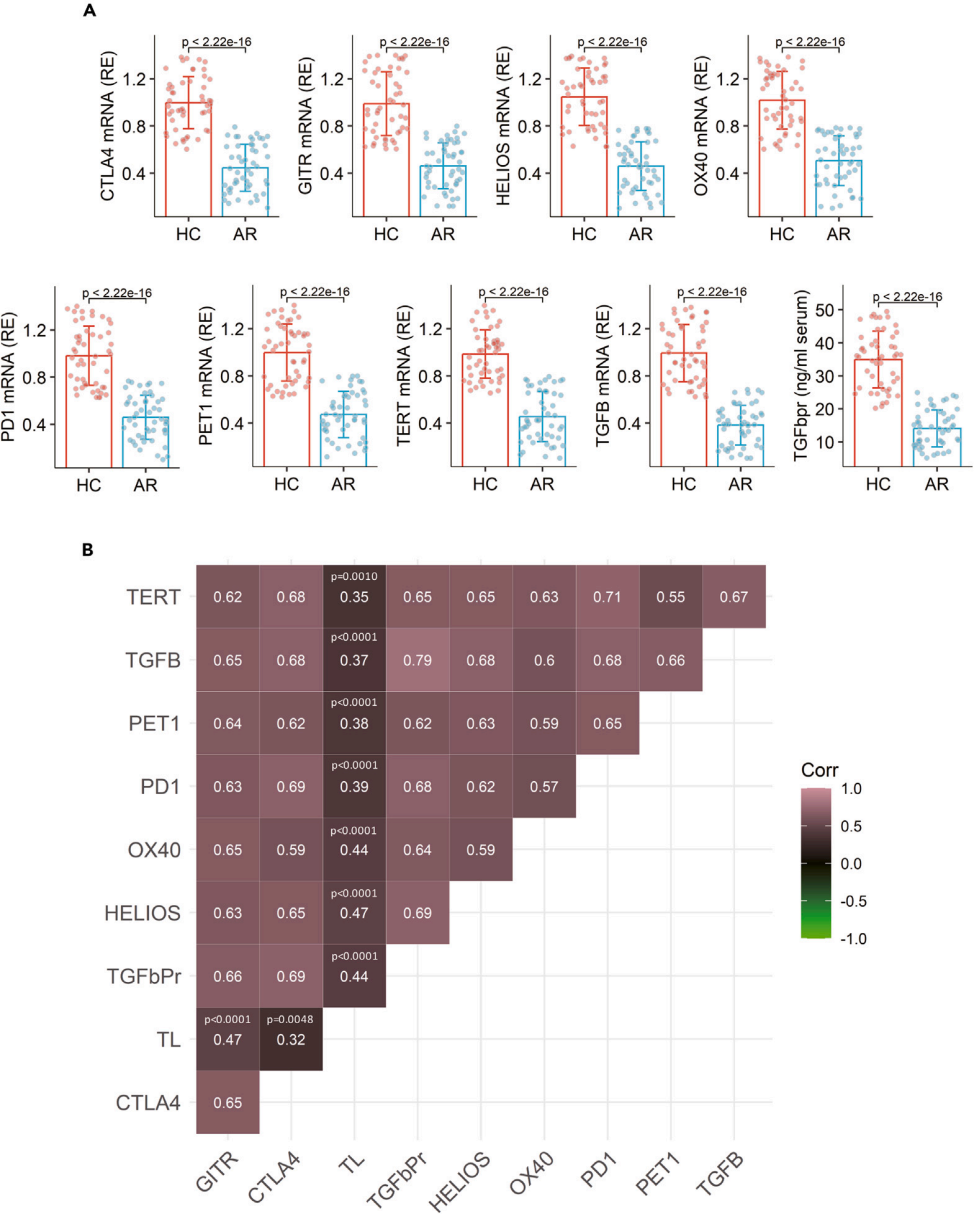
Association between immune regulatory molecules and TL/TEP1/TERT in Tregs PBMCs were prepared from blood samples collected from patients with AR (n = 50) and HC subjects (n = 50). Tregs were purified from PBMCs and analyzed by RT-qPCR. (A) Boxplots show mean ± SD of mRNA levels of indicated molecules in Tregs from 50 samples per group. (B) A heatmap shows positive correlation between indicated molecules. Statistics: Mann-Whitney test (A) and Spearman correlation coefficient assay (B). p values are presented in figures where appropriate. Each dot in bars presents one sample (tested in triplicate). The experiments were repeated 3 times. Abbreviations: HC: Healthy control. AR: Allergic rhinitis. PBMC: Peripheral blood mononuclear cell. Treg: Regulatory T cell. TL: Telomere length. TERT: Telomerase reverse transcriptase), TEP1: Telomerase protein 1. TGFb: TGF-β. TGFbpr: TGF-β protein.

We then assessed the amounts of the recognized immune regulatory molecules^22^ in Tregs by RT-qPCR. We found that the mRNA levels of transforming growth factor β (TGF-β), OX40 (Tumor necrosis factor receptor superfamily member 4), PD-1 (Programmed cell death protein 1), GITR (Glucocorticoid-induced TNFR-related protein), CTLA4 (Cytotoxic T-lymphocyte-associated antigen 4), and Helios in Tregs were significantly lower in the AR group than those in the HC group (Figure 2A). Positive correlation was detected between TL, TERT, TEP1, and the mRNA levels of the immune regulatory mediators in Tregs (Figure 2B). Serum TGF-β quantity was lower in the FA group than that in the HC group, which was also positively correlated with TL/TERT/TEP1 mRNA in Tregs (Figure 2). The results implicate that the lower activities of TERT, TEP1, and TL may be associated with the reduced immune-suppressive molecules in Tregs of patients with AR.

### The immune-suppressive functions of Tregs are associated with TL/TERT/TEP1

**Figure 3:**
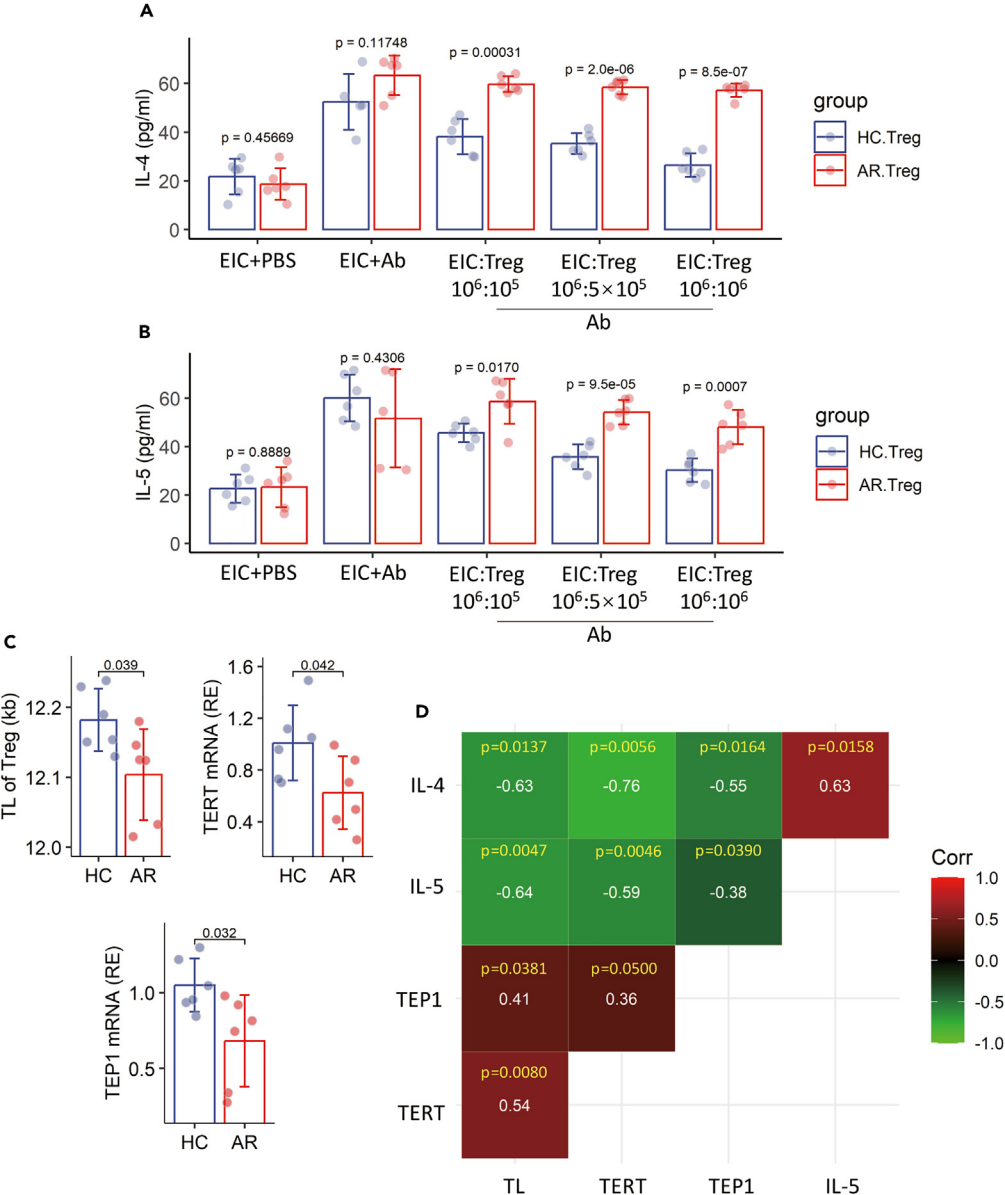
The immune-suppressive capacity is associated with TL in Tregs Tregs were isolated from blood samples collected from HC subjects (n = 6) and AR patients (n = 6). EICs were isolated from blood samples collected from HC subjects and labeled with CFSE. Tregs and EICs were cocultured at indicated ratios in the presence or absence of anti-CD3/CD28 Ab overnight. Culture supernatant was analyzed by ELISA. (A and B) Bars show mean ± SD of the amounts of IL-4 (A) and IL-5 (B) in supernatant. (C) Bars show mean ± SD of the mRNA levels of indicated molecules in Tregs (the group with EIC:Treg = 10^6^:10^6^ cell/mL). (D) Correlation between indicated molecules. The coefficients are presented in each square. Statistics: Student’s *t* test. p values are presented in figures where appropriate. Each sample was tested in triplicate. The experiments were repeated three times. Abbreviations: HC: Healthy control. AR: Allergic rhinitis. PBMC: Peripheral blood mononuclear cell. Treg: Regulatory T cell. Teff: Effector T cells (CD4^+^ CD25¯ T cell). TL: Telomere length. TERT: Telomerase reverse transcriptase), TEP1: Telomerase protein 1. FCM: Flow cytometry.

Interleukin (IL)-10 and TGF-β are the immune regulatory molecules of Tregs. The data reported earlier suggest that TL may be associated with the immune regulatory functions of Tregs. To test this, the immune-suppressive functions of Tregs collected from AR patients and HC subjects were assessed by the CFSE-dilution assay. The results showed that the immune-suppressive functions of Tregs from AR patients were weaker as compared with those of HC subjects (Figures 3A and 3B). Further analysis showed a negative correlation between the immune-suppressive functions and TL/TERT mRNA/TEP1 mRNA in Tregs (Figures 3C–3D). The results suggest that the status of TL/TERT/TEP1 is associated with Treg’s immune-suppressive functions.

### Sensitization reduces the levels of TL/TERT/TEP1 in Tregs

**Figure 4:**
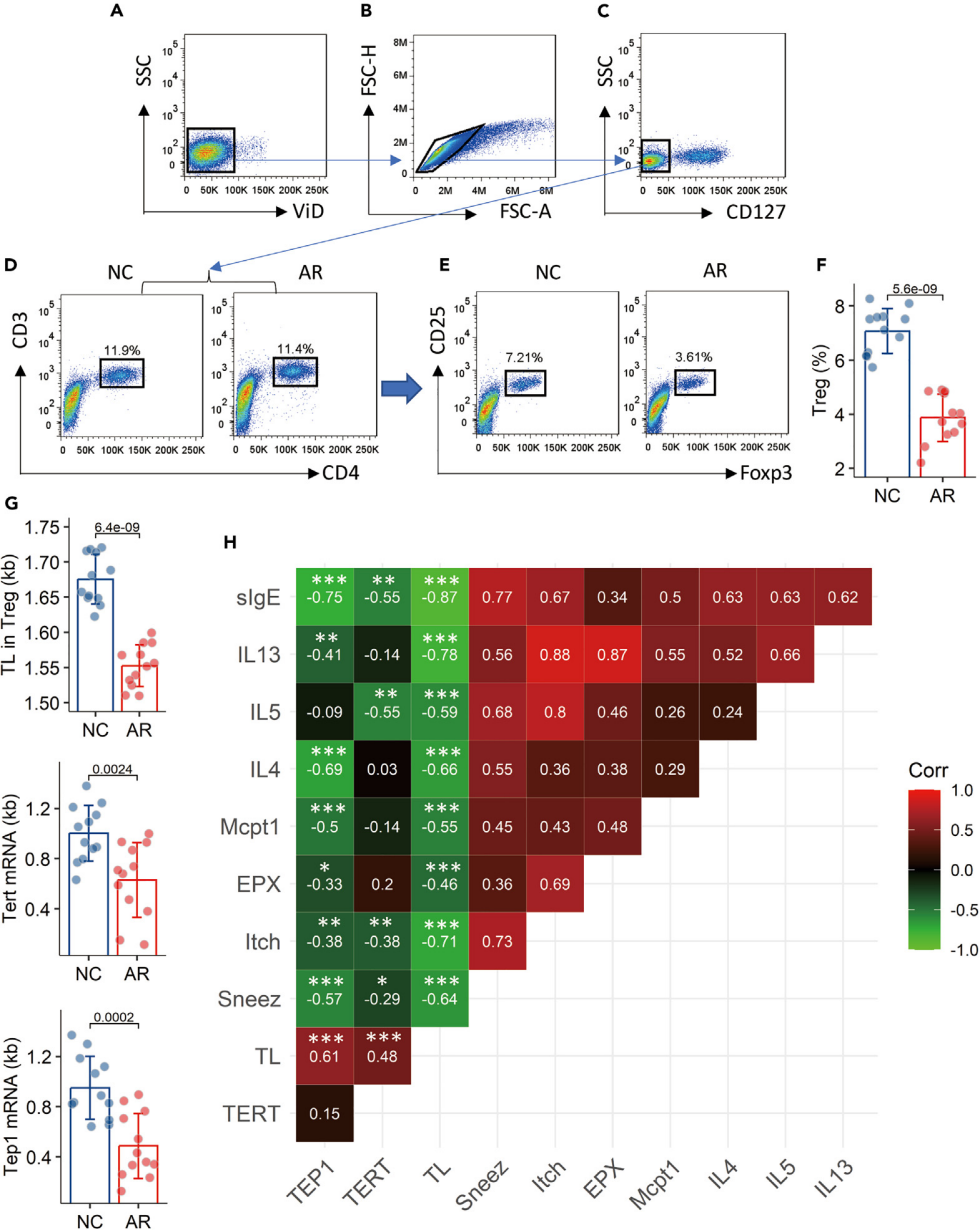
Sensitization suppresses the levels of TL, TERT, and TEP1 in Tregs of AR mice (A‒E) FCM plots show the gating strategy of isolating Tregs from AMCs. The boxplots show Treg counts in AMCs from 6 mice per group. (F) The measurements of TL, TERT mRNA, and TEP1 mRNA in Tregs. (G) The heatmap shows correlation coefficient between TL/TERT/TEP1 in Tregs and the AR response (the data are presented in Figure S2). Each group consists of 6 mice. Data of bars are presented as mean ± SD. Statistics: Student’s *t* test (F, G) and Pearson correlation coefficient assay (G). The experiments were repeated three times. Abbreviations: NC: Naive control mice. AR: Allergic rhinitis mice. FCM: Flow cytometry. AMC: Airway mononuclear cell. (H) A heatmap shows correlation between telomerases in Tregs and AR responses. The coefficients and their statistical results are prsented in the heatmap.

We then established a mouse model of AR (Figure S1 in supplemental information). Sensitized mice showed the AR response, including nasal itch and sneezing (the AR clinical symptoms), increase in eosinophil peroxidase (EPX), mouse mast cell protease-1 (Mcpt1), Th2 cytokines (IL-4, IL-5, IL-13) in nasal lavage fluids (NLF), and increase in serum specific IgE (sIgE) (Figure S2). The frequency of Treg in the airway mononuclear cells was lower in the sensitized mice than that of naive control (NC) mice (Figures 4A–4E). We found that TL was shorter in Tregs of AR mice as compared to that of NC mice. The levels of TERT and TEP1 in Tregs were lower in the AR group than those in the NC group (Figure 4F). Correlation assay results showed negative correlation between TL/TERT/TEP1 in Tregs and the AR response (Figure 4G). The results suggest that the activities of TL/TERT/TEP1 in Tregs are associated with the pathogenesis of airway allergy.

### The ER stress signaling pathway of PERK-eIF2a is associated with the regulation of telomerase

**Figure 5:**
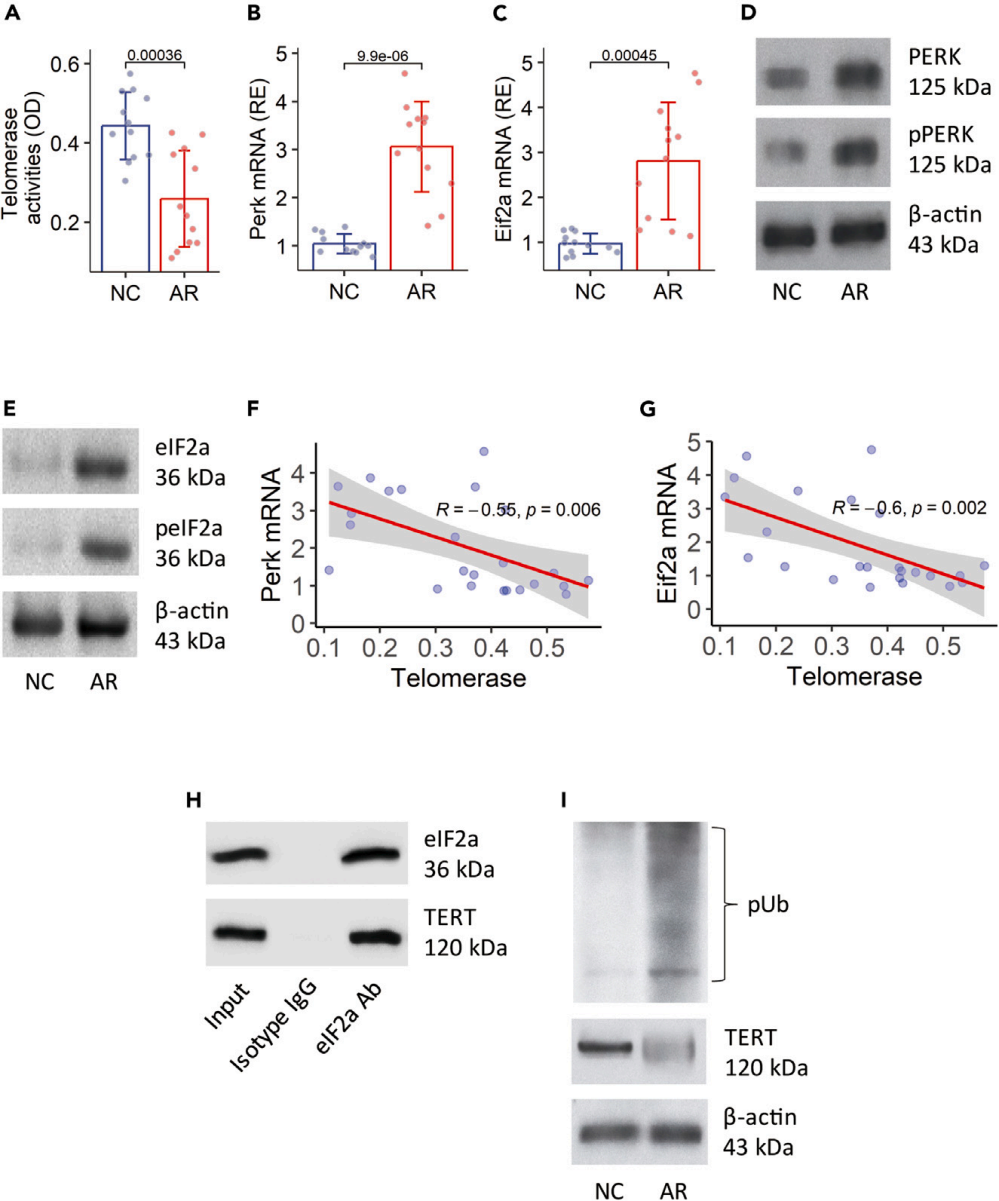
Assessment of the role of eIF2a in regulation of the telomerase activity (A‒E) Tregs were isolated from the airway tissues of NC mice (n = 12) and AR mice (n = 12). (A) The telomerase activity in Tregs. (B and C) The mRNA levels of *Perk* and *Eif2a* in Tregs. (D and E) The protein levels of PERK and eIF2a in Tregs. (F and G) Negative correlation between the telomerase activity and the mRNA levels of *Perk*/*Eif2a* in Tregs. (H) IP results show a complex of eIF2a and TERT detected in protein extracts from Tregs. (I) Immunoblots show TERT ubiquitination in Tregs. The eIF2a Ab was the IP Ab. The data of bars are presented as mean ± SD. Each dot in bars presents one sample. Statistics: Student’s *t* test (A‒C) and Pearson correlation coefficient test (F, G). The data of D, E, H, and I are from one experiment, which represent three independent experiments with pooled samples per group. Abbreviations: NC: Naive control mice. AR: Allergic rhinitis mice. PMA: Phorbol myristate acetate (50 ng/mL; a cell activator).

Since TL is regulated by telomerase, we assessed the activity of telomerase in Tregs. The results showed that the telomerase activity in Tregs was detectable in NC mice but was significantly lower in AR mice (Figure 5A). This phenomenon was also found in Tregs isolated from AR patients (Figure S3). The data of Figure 1 show that the levels of the *PERK* and *EIF2A* genes were elevated significantly in Tregs of AR patients. Such a phenomenon was also found in Tregs isolated from the airway tissues of AR mice (Figures 5B–5E). The mRNA levels of *Perk* and *Eif2a* were negatively correlated with the telomerase activity in Tregs (Figures 5F and 5G). The fact suggests that these ER stress-associated molecules may be involved in the regulation of telomerase in Tregs. To test this, protein extracts from AR Tregs were prepared and analyzed by immunoprecipitation (IP) using the Ab of eIF2a as the IP Ab. A complex of eIF2a and TERT was detected in AR Tregs (Figure 5H). Ubiquitination was detected in TERT protein of AR Tregs. TERT protein quantity was significantly less in samples from AR Tregs (Figure 5I). The results indicate that the sensitization induces ER stress in Tregs. The ER stress-associated proteins, eIF2a in this case, induce TERT protein ubiquitination and degradation and, thus, reduce the quantity of TERT in Tregs.

### Inhibition of eIF2a mitigates experimental AR through regulating telomerase activity in Tregs in the airway tissues

**Figure 6:**
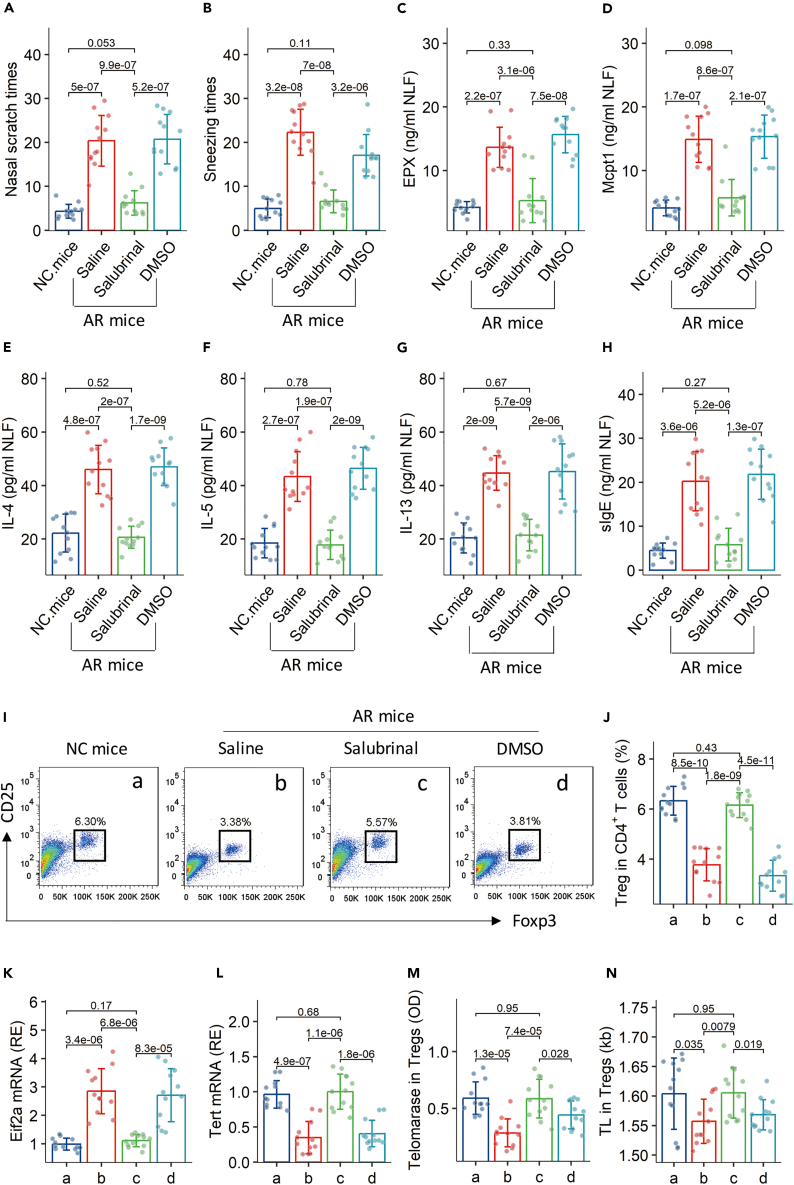
Assessment of the effects of an eIF2a inhibitor on mitigating experimental AR An AR mouse model was established using the DME protocol. (A‒H) The AR response, including AR clinical symptoms (nasal itch and sneezing; A and B), allergic mediators (EPX and Mcpt1; [C and D]) and Th2 cytokines (IL-4, IL-5, IL-13; [E‒G]) in NLF, and serum sIgE (H). (I and J) AMCs were isolated from the airway tissues and analyzed by FCM. Gated FCM plots show Treg counts in CD4^+^ T cells (the gating strategy is the same as Figure 4). Boxplots show Treg counts of 6 mice per group. (K‒N) Tregs were purified from AMCs. (K and L) mRNA levels of *Eif2a* and *Tert* in Tregs. (M) Telomerase activity. (N) TL in Tregs. The data of bars are presented as mean ± SD of indicated items from 12 mice per group. Each dot in bars presents one sample. Statistics: ANDME followed by the Bonferroni test. The group labels of J–N are the same as those of I. The experiments were repeated three times. Abbreviations: NC: Naive control. AR: Allergic rhinitis. Salubrinal: An inhibitor of eIF2a in nasal instillations (20 μM). FCM: Flow cytometry. AMC: Airway mononuclear cell. DME: DME. TL: Telomere length. Treg: Regulatory T cell. NLF: Nasal lavage fluid. DMSO: Dimethyl sulfoxide (the solvent of Salubrinal; 0.2 μg DMSO/mL in nasal instillations).

An AR mouse model was established with the DME protocol (Figure S1) with or without treating mice with Salubrinal (an inhibitor of eIF2a). AR mice showed AR response, including AR clinical symptoms (nasal itch and sneezing) (Figures 6A and 6B), increase in allergic mediators (EPX and Mcpt1), Th2 cytokines (IL-4, IL-5, and IL-13), and sIgE in NLF (Figures 6C–6H). The treatment with Salubrinal-containing nasal instillations efficiently suppressed the AR response (Figures 6A–6H), increased the frequency of Tregs in the airway tissues (Figures 6I and 6J), suppressed the expression of *Eif2a* (Figure 6K), and restored the telomerase activity and TL in Tregs (Figures 6L–6N). The results suggest that regulating the telomerase and TL in Tregs has a translational potential for the treatment of airway allergy.

## Discussion

The present study revealed that TL in Tregs was associated with the pathogenesis of AR. TL was shorter in peripheral Tregs of AR patients than that in HC subjects. Immune regulatory molecules, including IL-10 and TGF-β, were positively correlated with TL in Tregs. The levels of IL-10 and TGF-β in Tregs reflect the immune-suppressive capacity. The data also show that TL in Tregs was positively associated with the immune-suppressive ability. The phenomenon was reproduced in an AR mouse model. Tregs isolated from the airway tissues of AR mice showed shorter TL in Tregs compared with that of NC mice. TL was negatively correlated with the AR response. By RNA-seq, we found high levels of ER stress molecules, including PERK and eIF2a, in Tregs. This was also observed in Tregs isolated from AR mice. eIF2a could suppress telomerase. Administration of Salubrinal in AR mice restored telomerase activity, TL, and the number of Tregs in the airway tissues and mitigated experimental AR.

The data show that TL in Tregs is associated with the pathogenesis of AR. TL is a biomarker of the cell destiny.^23^ It becomes shorter in parallel to the number of cell dividing times.^23^ The changes of TL in tissue cells may not be obvious and easily detected.^24^ However, it can be measurable in those cells that divide frequently. Immune cells divide frequently, especially during immune response.^24^ Our data show that TL in Tregs was shorter in AR patients as compared to that of HC subjects. As TL is a critical biomarker of the cell destiny,^23^ the results implicate a link between TL and events associated with Treg activities.

The data show that TL/telomerase of Tregs in AR patients is positively correlated with AR-associated parameters or the AR response. It is well known that the number of Treg is reduced in AR patients.^11^ In line with these pioneering studies, the present data also show the reduction of Treg number in AR patients. A positive correlation was detected between the number of Tregs in the airway tissues, the Treg immune-suppressive functions, and the levels of TL, TERT, and TEP1 in Tregs. The results implicate a link between the abnormal TL in Tregs and Treg numbers/functions. Other investigators found that abnormal TL in the cell was associated with the pathogenesis of many diseases. For example, in cancer development, cancer cells need the telomere DNA maintained properly to counteract telomere shortening. This can protect telomeres from DNA damage and avoid telomere-mediated apoptosis or senescence.^23^ Abnormal TL was substantially associated with the risk of suffering from systemic lupus erythematosus in a European population.^25^ Patients with life-course-persistent asthma had shorter TL in leukocytes than healthy controls.^24^ The aforementioned information suggests that pathogenic condition may be associated with TL shortening in the cell.

Thus, elucidating factors inducing TL abnormal shortening is of significance. By sensitizing mice with the DME protocol, an AR mouse model was established in the present study. We found TL was shortened in Tregs in AR mice. TL was positively correlated with AR-associated parameters. The results suggest that the pathologic factors of sensitization cause TL shortening. We found that the ER stress-associated molecules, PERK and eIF2a, are uniquely upregulated in Tregs of AR patients as well as AR mice. Others also found that the signaling of the PERK-eIF2a pathway was elevated in experimental airway allergy.^26^ eIF2a is a global protein suppressor. Previous reports indicate that eIF2a reduces p53 levels by promoting its ubiquitination.^27^ Current data show that eIF2a promotes the degradation of TERT in AR Tregs. TERT is the catalytic subunit of the enzyme telomerase that is essential for telomerase activity.^28^ Thus, TL shortening in AR Tregs may be attributed to eIF2a-induced TERT degradation. In fact, eIF2a is a protein translational initiation factor in its canonical functions. More in-depth studies about the mechanism by which eIF2a induces TERT degradation need to be further conducted.

By using an inhibitor of eIF2a, Salubrinal, the experimental AR response was efficiently mitigated. Salubrinal has been used in the studies of ER stress inhibition. It can improve cardiac hypertrophy,^11^ osteoporosis,^12^ arthritis,^13^ and inhibition of TNF-α-triggered NF-κB activation.^14^ Current data indicate that administration of Salubrinal also can attenuate experimental airway allergy by improving the abundance of Tregs in the airway tissues. The underlying mechanism is that Salubrinal improves the telomerase activities, which further restore Tregs’ functions in the local tissues of AR mice.

In summary, TL is shorter in Tregs of subjects with AR, which is positively correlated with AR response. Sensitization shortens TL of Tregs in the airway tissues of AR mice. ER stress-associated molecules, PERK and eIF2a, can be increased in Tregs in response to sensitization. Inhibition of eIF2a can improve the status of telomerase and TL in Tregs and attenuate experimental AR in mice.

### Limitations of the study

Some data of the paper were generated from animal model studies. Large abundant cells obtained from the airway tissues of AR patients may further enrich our understanding on the role of ER stress in the regulation of Treg cells.

## STAR★Methods

### Key resources table

**Table:** 

REAGENT or RESOURCE	SOURCE	IDENTIFIER
**Antibodies**		

eIF2a	Santa Cruz Biotech	Clone#: 3A7A8, Cat#: sc-517214
TERT	Santa Cruz Biotech	C-12, sc-377511
TERT	Bios Antibodies	bs-20771R
TEP1	abcam	ab64189
PERK- phospho T982	abcam	ab192591
PERK	abcam	ab229912
elF2a (phospho S52)-N-terminal	abcam	ab227593

**Critical commercial assays**		

DME-specific IgE ELISA kit	Huabang Biotech	-
Mcpt1 ELISA kit	R&D Systems	AF5146
IL-4 ELISA kit	R&D Systems	M4000B, D4050
IL-5 ELISA kit	R&D Systems	M5000, D5000B
IL-13 ELISA kit	R&D Systems	M1300CB, D1300B
EPX ELISA kit	Jianglai Biotech	-

**Chemicals, peptides, and recombinant proteins**		

Salubrinal	Solarbio Life Science	

**Software and algorithms**		

R v4.3.1	R Development Core Team, 2022	https://mirrors.bfsu.edu.cn/CRAN/
FlowJo v10	BD	https://www.flowjo.com/
BD FACSCanto™ Clinical Software	BD	https://www.bdbiosciences.com/en-eu/products/software/instrument-software/bd-facscanto-clinical-software

### Resource availability

#### Lead contact

Further information and requests for resources and reagents should be directed to and will be fulfilled by the lead contact: Pingchang Yang (pcy2356@szu.edu.cn).

#### Materials availability

All new reagents are available in our lab for non-commercial research.

#### Data and code availability

All the data are included in this paper. This paper does not have specific code. Any additional information required to reanalyze the data reported in this paper is available from the lead contact upon request.

### Experimental model and subject details

#### Ethics statement

The study protocol was approved by the human ethics committee and the animal ethics committee at our institution. A written informed consent was obtained from each human subject. All animal experiments were conducted in accordance to the ARRIVE guidelines.

#### Human subjects

Patients with perennial allergic rhinitis (AR) were enrolled into this study at our hospital. To minimize the aging factor in TL assessment, subjects with age between 20 and 30 years were included. The diagnosis of AR was made by our physicians following the established AR diagnosis procedures that can be found elsewhere. In brief, patients had AR history more than two years, serum specific IgE (sIgE) and skin prick test (SPT) positive. Subjects with any of the following conditions were excluded: severe organ diseases, autoimmune diseases, cancers, under treatment with immune suppressive agents for any reasons. The age- and gender-matching healthy control (HC) subjects were also recruited.

### Method details

#### Preparation of peripheral blood mononuclear cells (PBMC)

Blood samples (20 ml per person) were collected from each human subject through the ulnar vein puncture. PBMCs were isolated from the blood samples by gradient density centrifugation.

#### Measurements of TL in Tregs

Total DNA was isolated from Tregs and quantified using a NanoDrop SD-1000 spectrophotometer. Relative telomere length in Treg DNA was measured and presented as the telomere/single-copy-gene (T/S) ratio (the proportional telomere to the average telomere length in a cell) following published procedures.^29^

#### Assessment of telomerase activity, cytokines and specific IgE (sIgE) by enzyme-linked immunosorbent assay (ELISA)

Telomerase activity was assessed using TeloTAGGG Telomerase PCR ELISA kit (Roche Diagnostics GmbH) following the manufacturer’s instructions. Cytokines and sIgE were assessed by ELISA using commercial ELISA kits following the manufacturer’s instructions.

#### Assessment of immune suppressive functions of Treg

Effective immune cells (EICs) were cultured with Tregs at gradient ratio of 10^6^:10^5^, or 10^6^:5 × 10^5^, or 10^6^:10^6^, in the presence of CD3/CD28 Abs (5 μg/ml each) overnight. The supernatant was analyzed by ELISA to measure the amounts of IL-4 and IL-5, which were used as indicators of Tregs’ immune suppressive functions.

#### Flow cytometry (FCM)

In the surface staining, cells were incubated with fluorescence labeled Abs (Ab types are detailed in figures) or isotype IgG for 30 min at 4°C. After washing with FCM buffer (phosphate buffered saline, PBS, containing 2% bovine serum albumin, BSA) 3 times, cells were analyzed with a flow cytometer (BD FACSCanto II). In the intracellular staining, cells were fixed with 1% paraformaldehyde (containing 0.05% Triton X-100) for 1 h. After washing with PBS 3 times, cells were processed with the procedures of the surface staining. In the case of staining both surface markers and intracellular markers, surface markers were stained first; cells were then processed with the intracellular staining procedures. The data are analyzed with Flowjo software (TreeStar Inc., Ashland, OR). The data from isotype IgG staining were used as a gating reference.

#### Isolation of immune cells from PBMCs

PBMCs were stained with fluorescence labeled Abs of CD3, CD4, CD25, and CD127 for 30 min at 4°C. With FCM cell sorting, CD3^+^ T cells were gated, from which the CD127^+^ cells were gated out. Then, the CD4^+^ CD25^+^ CD127¯ cells or CD4^+^ CD25¯ T cells were sorted and used as Tregs. The Treg-excluded PBMCs were used as effective immune cells (EICs) in experiments.

#### Real-time quantitative RT-PCR (RT-qPCR)

Total RNA samples were extracted from Tregs using the TRIzol reagents. The cDNA was synthesized using the RNA samples with a reverse transcription kit following the manufacturer’s instructions. The samples were amplified in a qPCR device (Bio Rad CFX96) with a SYBR Green Master Mix kit in the presence of relevant primers, including PERK (homo: ctcacaggcaaaggaaggag and aacaactccaaagccaccac; mus: cggagacagtgtttggctta and gctttttcccatcattctcg), EIF2A (homo: tctgaggggacaaatggaag and attctgcatttgatggcaca; mus: atgatgtgccatcaaatgga and catgcagtttggaattggtg), TERT (homo: cgtggtttctgtgtggtgtc and ccttgtcgcctgaggagtag; mus: agggtaagctggtggaggtt, gatgctctgctcgatgacaa), TEP1 (homo: cccaagtccctgaactgtgt and acattgaaggccaaggtacg; mus: gagcaccttggagcaagaac and agggtttccactgtgactgg), IL-10 (ccaagctgagaaccaagacc and aaggcattcttcacctgctc), TGFB1 (gggactatccacctgcaaga and cctccttggcgtagtagtcg). The results were calculated with the 2^-ΔΔCt^ method and presented as relative expression (RE).

#### Western blotting

Proteins were extracted from Tregs, separated by SDS-PAGE, and transferred onto a PVDF membrane. The membranes were blocked by incubating with 5% skim milk for 30 min, stained with Abs of interest at a concentration of 200 ng/ml for 2 h, followed by incubating with HRP-labeled second Abs at a concentration of 20 ng/ml for 2 h. Washing with TBST (Tris-buffered saline containing 0.05% Tween 20) 3 times after incubating with Abs. The immunoblots on the membrane were developed with the enhanced chemiluminescence and recorded by photographing in an imaging station (UVP, Cambridge, UK).

#### Immunoprecipitation (IP)

Proteins were extracted from Tregs, and precleared by incubating with protein G agarose beads for 2 h. The beads were discarded by centrifugation. The samples were incubated with an eIF2a Ab (0.5 μg/ml) overnight. The immune complexes in samples were adsorbed by protein G agarose beads. The beads were collected by centrifugation. Proteins on the beads were eluted and analyzed by Western blotting. The procedures of IP were performed at 4°C.

#### RNA sequencing (RNAseq)

RNA was extracted from Tregs, and sent to a biotech company (Yigene, Shenzhen, China) to be analyzed with RNAseq. The RNAseq procedures were performed by professional staff of the company. The data were analyzed by the technical staff. The differentially expressed genes (DEGs) were expressed as volcano plots. The gene oncology (GO) annotation results were expressed by barplots.

#### Establishment of an AR mouse model

The animal experimental procedures were approved by the Animal Ethics Committee at Shenzhen University (approve#2022-091). The experiments were conducted in accordance with the ARRIVE guidelines. Male BALB/c mice (6-8-week-old) were purchased from Guangdong Experimental Animal Center (Foshan, China). Mice were maintained in a specific pathogen free facility at Shenzhen University with accessing food and water freely. To establish an AR animal model, mice were treated with the procedures depicted in Figure S1 in supplemental information. Briefly, mice were subcutaneously injected with DME (0.1 mg/mouse) on the back skin on day 1 and day 7, respectively. Mice were then boosted with nasal instillations (20 μl/nostril containing DME 5 mg/ml) daily from day 9 to day 22. On day 23, mice were challenged with nasal instillations (20 μl/nostril containing DME 50 mg/ml).

### Quantification and statistical analysis

The data are presented as median (IQR) or mean ± SD. The difference between two groups was determined by Student’s *t*-test or the Mann Whitney test. ANDME followed by Dunnett’s test or Bonferroni test was conducted for multiple comparisons. Correlation coefficient assay was performed to determine the correlation between two groups. P<0.05 was set as a significant criterion.

## Supplemental information

Document S1. Figures S1‒S3
