# Supplementary material for: Undersized telomeres in regulatory T cells link to the pathogenesis of allergic rhinitis
Source: iScience. 2023 Dec 10;27(1):108615. doi: 10.1016/j.isci.2023.108615 (PMC10777067; doi:10.1016/j.isci.2023.108615)
Supplement: Document S1. Figures S1‒S3 [file mmc1.pdf]

iScience, Volume *27*

Supplemental information

Undersized telomeres in regulatory T cells link to the pathogenesis of allergic rhinitis

Jinmei Xue, Zhizhen Liu, Yun Liao, Xiwen Zhang, Yu Liu, Lihua Mo, Rui Dong, Qiang Li, Xizhuo Sun, Jun Xie, and Pingchang Yang

Supplemental materials

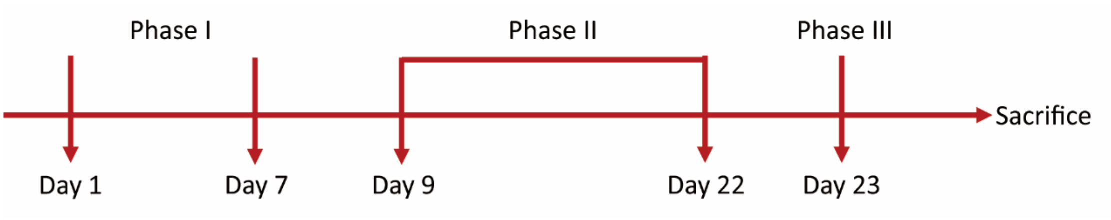

Figure S1. A schematic of the "DME" protocol using to establish an AR mouse model (Related to Figure 4). Phase I: Mice (12 mice per group) were subcutaneously injected with DME (0.1 mg/mouse) on the back skin on day 1 and day 7, respectively. Phase II: Mice were boosted with nasal instillations (20 μl/nostril containing DME 5 mg/ml) daily from day 9 to day 22. Phase III: Mice were challenged with nasal instillations (20 μl/nostril containing DME 50 mg/ml).

Abbreviations: DME: Dust mite extracts. AR: Allergic rhinitis.

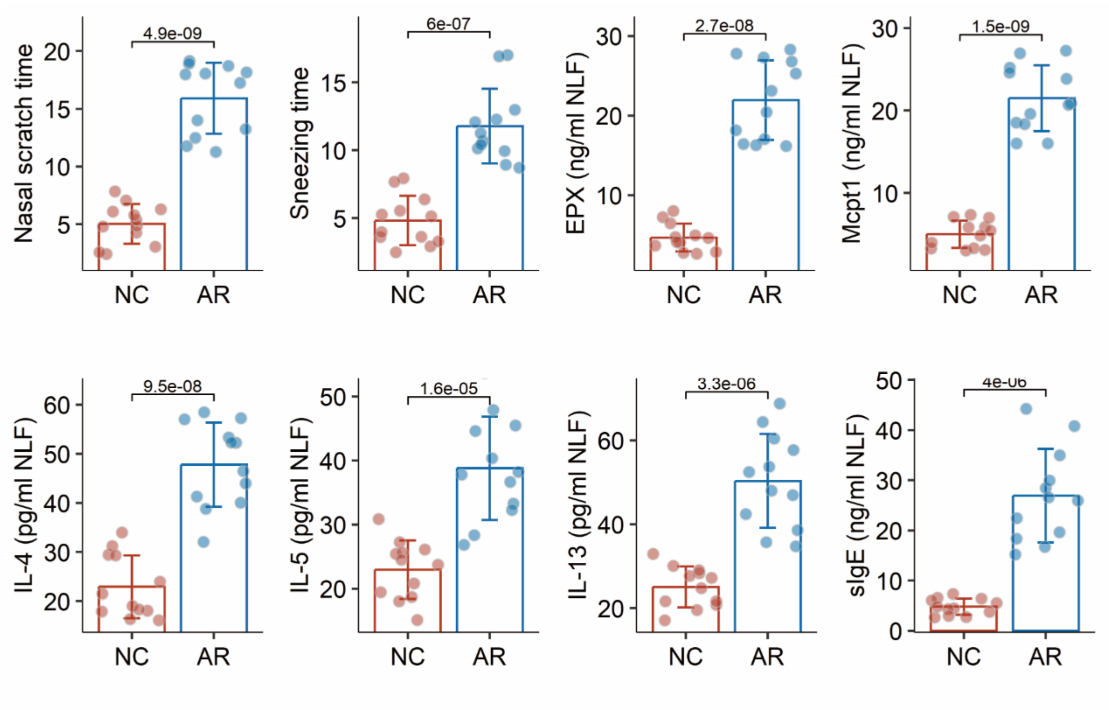

Figure S2. Establishment of an AR mouse model (Related to Figure 4). Mice were treated with the DME protocol as depicted in Fig. S1. Boxplots show the AR response, including AR clinical symptoms (nasal scratch times and sneezing), allergic mediators (EPX and Mcpt1), Th2 cytokines (IL-4, IL-5, and IL-13) and sIgE in NLF. The data of boxplots are presented as mean ± SD of 12 mice per group. Each dot in bars presents one sample (tested in triplicate). Statistics: Student *t*-test. *p* values are presented in figures where appropriate.

Abbreviations: NC: Naïve control mice. AR: Allergic rhinitis mice. DME: DME. EPX: Eosinophil peroxidase. Mcpt1: Mouse mast cell protease 1. sIgE: Mite specific IgE.

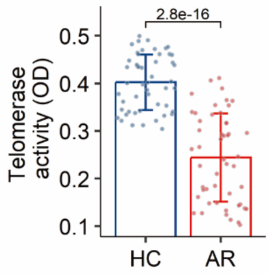

Figure S3. Assessment of telomerase activity in Tregs of human subjects (Related to Figure 5). Tregs were isolated from blood samples collected from HC subjects (n = 50) and AR patients (n = 50). Protein extracts were prepared from Tregs of each sample, and pooled. Bars show mean ± SD of telomerase activity (OD value) from 50 samples per group. Statistics: Mann Whitney test.
